# Knocking down gene expression for growth hormone-releasing hormone inhibits proliferation of human cancer cell lines

**DOI:** 10.1038/sj.bjc.6604386

**Published:** 2008-05-27

**Authors:** N Barabutis, A V Schally

**Affiliations:** 1 Veterans Affairs Medical Center and South Florida Veterans Affairs Foundation for Research and Education, Miami, FL 33125, USA; 2 Department Of Pathology and division of Hematology/Oncology, Department of Medicine, University of Miami, Miller School of Medicine, Miami, FL 33125, USA

**Keywords:** breast cancer, lung and prostate cancer, GHRH

## Abstract

Splice Variant 1 (SV-1) of growth hormone-releasing hormone (GHRH) receptor, found in a wide range of human cancers and established human cancer cell lines, is a functional receptor with ligand-dependent and independent activity. In the present study, we demonstrated by western blots the presence of the SV1 of GHRH receptor and the production of GHRH in MDA-MB-468, MDA-MB-435S and T47D human breast cancer cell lines, LNCaP prostate cancer cell line as well as in NCI H838 non-small cell lung carcinoma. We have also shown that GHRH produced in the conditioned media of these cell lines is biologically active. We then inhibited the intrinsic production of GHRH in these cancer cell lines using si-RNA, specially designed for human GHRH. The knocking down of the GHRH gene expression suppressed the proliferation of T47D, MDA-MB-435S, MDA-MB-468 breast cancer, LNCaP prostate cancer and NCI H838 non-SCLC cell lines *in vitro*. However, the replacement of the knocked down GHRH expression by exogenous GHRH (1–29)NH_2_ re-established the proliferation of the silenced cancer cell lines. Furthermore, the proliferation rate of untransfected cancer cell lines could be stimulated by GHRH (1–29)NH_2_ and inhibited by GHRH antagonists MZ-5-156, MZ-4-71 and JMR-132. These results extend previous findings on the critical function of GHRH in tumorigenesis and support the role of GHRH as a tumour growth factor.

Dysfunction of cell differentiation and cell cycle regulation define and promote carcinogenesis. Growth hormone-releasing hormone (GHRH) was first isolated from human pancreatic tumours and only subsequently identified in human and animal hypothalami (reviewed in Schally and Varga, 2006). The full intrinsic biological activity of GHRH is retained by the NH_2_-terminal 29 amino acid sequence. Growth hormone-releasing hormone is secreted by the hypothalamus and upon binding to the specific GHRH-Receptors (GHRH-R) on somatotrophs regulates the release of Growth Hormone (GH) from the anterior pituitary gland. In turn, GH stimulates the production of insulin-like growth factor-I (IGF-I), which functions as a cell cycle stimulator (Macaulay, 1992; Westley and May, 1995).

Growth hormone-releasing hormone-receptors (GHRH-R) is a class II G protein-coupled receptor and contains seven transmembrane domains (Mayo, 1992). Growth hormone-releasing hormone-receptor is homologous with the receptors for the vasoactive intestinal peptide (VIP), the pituitary adenyl cyclase activating polypeptide and calcitonin (Gaylinn *et al*, 1993). Recently, peptide receptors that mediate the effects of GHRH and its antagonists on tumours were identified. The isolation and sequencing of cDNAs which correspond to the tumoral GHRH receptor mRNA revealed that they are splice variants (SVs) of the pituitary GHRH receptors (pGHRH-R) (Rekasi *et al*, 2000).

Splice variant 1 of GHRH receptor is a functional receptor, which differs from the pGHRH-R only in the N-terminal extracellular domains. The first 89 amino acids of the pGHRH-R are replaced in SV-1 receptor by a different 25-amino acid sequence (reviewed in Schally and Varga, 2006). Some tumours also express pituitary type of GHRH receptor (Havt *et al*, 2005; Christodoulou *et al*, 2006). Besides its ligand-dependent activity, a ligand-independent activity of SV1 has also been demonstrated (Kiaris *et al*, 2003). A recent study showed the stimulation and proliferation of MCF-7 breast cancer cells after the transfection of SV1 (Barabutis *et al*, 2007). The expression of mRNA for GHRH and the presence of biologically or immunologically active GHRH were demonstrated in several established cancer cell lines and human tumours. Collectively, those data suggest that GHRH may function as a growth factor among a large class of mitogens involved in tumorigenesis. In an endeavour to develop new methods for cancer treatment, we developed the antagonists of GHRH (reviewed in Schally and Varga, 2006).

Growth hormone-releasing hormone antagonists suppress the *in vivo* growth of various experimental cancers such as prostatic (Zarandi *et al*, 2006; Stangelberger *et al*, 2007), mammary (Buchholz *et al*, 2007), ovarian (Chatzistamou *et al*, 2001), renal cell carcinomas (Halmos *et al*, 2000), small cell lung carcinomas (Hohla *et al*, 2006), pancreatic and colorectal carcinomas (Szepeshazi *et al*, 2000; Busto *et al*, 2002), endometrial (Engel *et al*, 2005), osteogenic sarcomas (Braczkowski *et al*, 2002) as well as malignant glioblastomas (Jaeger *et al*, 2005).

The inhibitory effect of antagonistic analogues of GHRH is exerted in part by endocrine mechanisms through the suppression of GHRH-evoked GH release from the pituitary, which, in turn results in the reduction of hepatic production of IGF-I and a decrease in the serum IGF-I levels. The anti-tumour effects of GHRH antagonists can be also mediated through direct mechanisms. One of these mechanisms is based upon the inhibition of the secretion of autocrine/paracrine IGF-I or IGF-II from the tumours, while probably the most important pathway involves the blockade of action of autocrine GHRH in tumours. The antitumour activity of our GHRH antagonists is especially important oncologically because of the wide expression of the intrinsic GHRH, pGHRH-R and SVs of GHRH-R in various cancers.

In the present study, we evaluated by western blot the expression of the SV-1 of GHRH-R and its GHRH ligand in human breast cancer (MDA-MB-468, MDA-MB-435S, T47D), prostate cancer (LNCaP) and non-SCLC (NCI H838) cell lines. We detected the GHRH produced in the conditioned medium of these cell lines. To further elucidate the role of GHRH in carcinogenesis, we knocked down the gene expression of GHRH. In addition, we examined the effect of GHRH and GHRH antagonists MZ-4-71, MZ-5-156 and JMR-132 at two dose levels on the proliferation rate of the cited cancer cell lines. Our results demonstrate the critical function of GHRH and its receptors in tumorigenesis.

## MATERIALS AND METHODS

### Peptides and chemicals

Growth hormone-releasing hormone antagonists JMR-132 [PhAc^0^, DArg^2^, Phe (4-C)^6^, Ala^8^, Har^9^, Tyr (Me)^10^, His^11^, Abu^15^, His^20^, Nle^27^, D-Arg^28^, Har^29^] human GHRH (1–29)NH_2_, MZ-5-156 [PhAc-Tyr^1^, D-Arg^2^, Phe (4-CI)^6^, Abu^15^, Nle^27^ hGHRH (1–28)Agm and MZ-4-71 [Ibu-Tyr^1^, D-Arg^2^, Phe (4-CI)^6^, Abu^15^, NIe^27^] hGHRH (1–28) Agm, where Abu is a-aminobutyric acid, Agm is agmatine, Har is homoarginine, Nle is norleucine, PhAc is phenylacetyl and Tyr(Me) is *O*-methyltyrosine were synthesized in our laboratory by solid phase methods (1,19). Growth hormone-releasing hormone (1–29)NH_2_ and GHRH antagonists were dissolved in 0.1% DMSO and diluted with incubation media.

### Cell culture

The cell lines (LNCaP, MCF-7, MDA-MB-468, MDA-MB-435s, T47D, and NCI-H838) were obtained from American Type Culture Collection (Manassas, VA, USA) and cultured at 37°C in a humidified 95% air/5% CO_2_ atmosphere. Breast cancer cell lines MCF-7, MDA-MB-468, MDA-MB-435s and T47D were cultured in DMEM supplemented with antibiotics/antimycotics and 10% FBS. Prostate cancer cell line (LNCaP) and non SCLC cell line NCI H838 were cultured in RPMI-1640 supplemented with antibiotics/antimycotics and 10% fetal bovine serum (FBS). The culture media were purchased from GIBCO (Carlsbad, CA).

### Protein isolation and western blot assay

The expression of GHRH and SV1 receptor was assessed by western blot in T47D, MDA-MB-435s, MDA-MB-468 breast cancer cell lines, LNCaP prostate cancer line and NCI H838 non-SCLC line. MCF-7 breast cancer cell line which does not produce either GHRH or SV1 receptor was used as negative control (Barabutis *et al*, 2007). The proteins were isolated from the cells using CelyticM Lysis Reagent (Sigma, St Louis, MO, USA) and the concentration of the isolated proteins was determined by Quickstart Bradford Protein Assay (Bio-Rad, Hercules, CA, USA) according to manufacturer's instructions. Protein matched samples (15 *μ*g per lane) were separated by electrophoresis 12.5 or 8–16% sodium dodecyl sulphate (SDS–PAGE Tris-HCL precast gels (Bio-Rad, Hercules, CA, USA). Electroblotting was used to transfer the proteins onto nitrocellulose membranes (Biorad, Hercules, CA, USA). The membranes were incubated for 3 h at room temperature in 5% non fat dry milk in phosphate-buffered saline (PBS) – 0.1% (v/v) Tween 20. The blots were then incubated at 4°C overnight with an affinity purified goat polyclonal antibody against a peptide mapping near the N terminus of GHRH of human origin (1 : 425) (cat no. 10280, Santa Cruz Biotechnology, Santa Cruz, CA, USA) and with rabbit antiserum to SV1 (1 : 2000) raised in our laboratory (Toller *et al*, 2004). The antiserum batch number for SV1 was JH 2317/5. The signal for the immunoreactive proteins was developed with peroxidase-conjugated secondary antibodies (Santa Cruz and Cell Signalling, Danvers, MA, USA) and visualised by exposure to chemiluminescence substrate (Amersham Biotechnologies, Piscataway, NJ, USA). The *β*-actin signal (1 : 1000, Santa Cruz) was used as control.

### Quantitative analysis of the immunoblot assay

The protein bands signals were quantified by Adobe Photoshop and normalised to *β*-actin signal. The intensity of the bands was equal to their mean value multiplied by their pixel value (absolute intensity). Relative intensity (RI) of each band is calculated by dividing its absolute intensity by the absolute intensity of the control band (*β*-actin). The percentage efficiency of the knocking down of the GHRH gene expression was calculated as {(R.I of the Pre-pro GHRH band of untransfected cells – RI of the pre-pro GHRH band of transfected cells)/RI of the Pre-pro GHRH band of untransfected cells} × 100.

### Radioimmunoassay of GHRH

Tumour cells (3 × 10^5^ cells) were seeded in 48-well petri dishes and allowed to attach for 24 h when the media were replaced by serum-free medium (SFM). Aliquots of 0.1 ml medium from MCF-7, T47D, MDA-MB-435s, MDA-MB-468 breast cancer cell lines, LNCaP prostate cancer cell line and NCI H838 non-SCLC cell line growing for 0, 24, 48 and 72 h were assayed for GHRH immunoreactivity. Growth hormone-releasing hormone was measured by using ([^125^I]-Tyr)-GHRH-]) (human) (Bachem, cat no. H-5028) as the labelled peptide and a rabbit antibody against GHRH (1–44)NH_2_ (Bachem, cat no. S 2027). This antibody crossreacts 100% with human GHRH (1–44)NH_2_ and 30% with human GHRH (1–29)NH_2_. Growth hormone-releasing hormone (1–44)NH_2_ (Bachem, cat no. H-3695) was used as a standard. The range was 1–128 pg per tube.

### Transfection

Small interfering (si) RNA designed specially for the inhibition of the human GHRH was used to knock down the GHRH gene expression of the T47D, MDA-MB-435s, MDA-MB-468 breast cancer cell lines, LNCaP prostate cancer cell line and NCI H838 non-SCLC cell line. A pool of three oligonucleotides especially designed for the inhibition of the human GHRH (sc39519, Santa Cruz Biotechnology) and cationic liposomes (Lipofectamine, Invitrogen, Carlsbad, CA, USA) were used to knock down the gene expression of the GHRH. The sequence of the sense strand (a) was GGUAUGCAGAUGCCAUCUUTT and the mRNA location is 92. The sequence of the sense strand (b) was CCAGUUAAUCCUCUCAUUUTT and its mRNA location is 376. The sequence of the sense strand (c) was CCAGUUAAUCCUCUCAUUUTT and its mRNA location is 434. RNA that does not lead to any specific degradation of any known cellular mRNA (control siRNA, sc37007, Santa Cruz Biotechnology) was used as control. The sequence of the sense strand was UUCUCCGAACGUGUCACGU?. One day before the transfection, the cells were plated in six-well plates in 2 ml of growth media without antibiotics to be 40% confluent on the day of transfection. For the transfection 40–60 nM siRNA was diluted in 0.25 ml Opti-MEM I Reduced Serum Medium (Invitrogen, Carlsbad, CA, USA) without serum and 5 *μ*l lipofectamine 2000 were diluted in an equal volume of Opti-MEM I. After incubation for 5 min at room temperature, the diluted oligomers were combined with the lipofectamine 2000 and incubated for 30 min also in room temperature. The oligomer-lipofectamine complexes were added to each well, which contained 2 ml of medium without antibiotics. The medium was changed 8 h after transfection. The cells were incubated at 37°C in an atmosphere of 5% CO_2_ and 95% air for 48–72 h after transfection and then assayed by western blot for gene knockdown.

### Cell proliferation rate assay

The rate of the cell proliferation was calculated by seeding 10 000 cells in six-well plates and after an incubation for 4 days counting them under light microscope using the trypan blue assay.

### Statistical analysis

These data are expressed as the mean±s.e.m. Statistical evaluation of the results was performed by the Student's *t*-test (two-tailed). *P*-values shown are against the control group.

## RESULTS

### Expression of growth hormone releasing hormone and splice variant 1 of the GHRH receptor in breast and prostate cancer and non-SCLC cell lines

**Figure 1:**
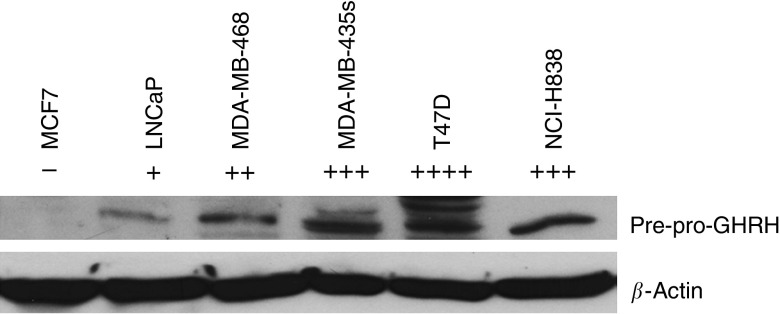
Western blot analysis of protein expression of GHRH in breast cancer (MCF-7, MDA-MB-468, MDA-MB-435s, T47D), lung cancer (NCI-H838) and prostate cancer (LNCaP) cell lines. MCF-7 breast cancer cell line was used as negative control. Signal intensity of the 45 kDa precursor GHRH protein is indicated by the symbol +. Protein levels were normalised to *β*-actin signal.

**Figure 2:**
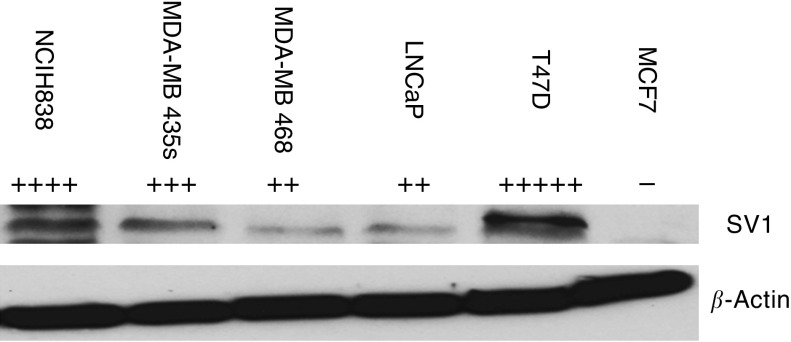
Western blot analysis of expression of SV1 in breast cancer (MCF-7, MDA-MB-468, MDA-MB-435s, T47D), lung cancer (NCI-H838) and prostate cancer (LNCaP) cell lines. MCF-7 breast cancer cell line was used as negative control. Signal intensity of GHRH protein is indicated by the symbol +. Protein levels were normalised to *β*-actin signal.

A band of 45 KDa which reflects the production of pre-pro GHRH (Othman *et al*, 2001) was detected in all the cancer cell lines examined. The results are shown in Figure 1. Pre-pro GHRH protein expression was the highest in T47D (R.I:0.464), followed by MDA-MB-435s (R.I:0.449), NCI H838 (R.I:0.437), MDA-MB-468 (R.I:0.312) and LNCaP (R.I:0.193). A band at 39.5 KDa which is consistent with the size of the SV1 receptor (Havt *et al*, 2005) was also detected in all the cancer cell lines examined. The results are shown in Figure 2. The levels of the SV1 GHRH-R protein were the highest in T47D (R.I:0.696), followed by NCI H838 (R.I:0.376), MDA-MB-435s (R.I:0.316), LNCaP (R.I:0.169), MDA-MB-468 (R.I:0.160).

### Detection of the secretion of growth hormone-releasing hormone in the conditioned medium of the cancer cell lines by radioimmunoassay

The concentration of the GHRH in samples from culture medium was measured by RIA. Significant amounts of GHRH were detected in the medium from T47D (1.518, 2.083, 0.266 ng ml^−1^), NCI H838(0.303, 0.442, 0.689 ng ml^−1^), MDA-MB-435s (0.675, 1.036, 1.442 ng ml^−1^), LNCaP(0.202, 0.218, 0.208 ng ml^−1^), and MDA-MB-46 ng ml^−1^ (0.637, 0.816, 0.649) cell lines after 24, 48 and 72 h respectively as shown in Table 1. Growth hormone-releasing hormone was not detected either in the cultured medium without cells or in the conditioned medium of the MCF7 breast cancer cell line.

### Inhibition of the GHRH gene expression in breast, prostate and non-SLCL cancer cell lines and its effect on proliferation

**Figure 3:**
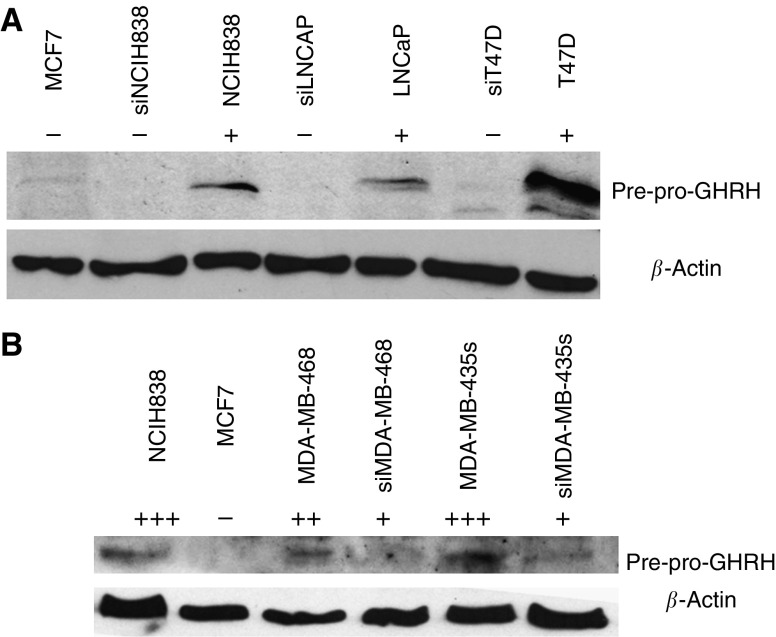
Western blot analysis of GHRH in cancer cell lines before and after the inhibition of the GHRH gene expression. The knocked down cells are marked as si. MCF-7 breast cancer cell line was used as negative control. Protein levels were normalised to *β*-actin signal. (**A**) The detection of the GHRH protein expression in breast (T47D), lung (NCI-H838) and prostate cancer (LNCaP) cell lines. (**B**) The detection of the GHRH protein expression in breast cancer (MDA-MB-468, MDA-MB-435s) cell lines. NCIH-838 cancer cell line was used as positive control. Signal intensity of GHRH protein is indicated by the symbol +. Protein levels were normalised to *β*-actin signal.

**Figure 4:**
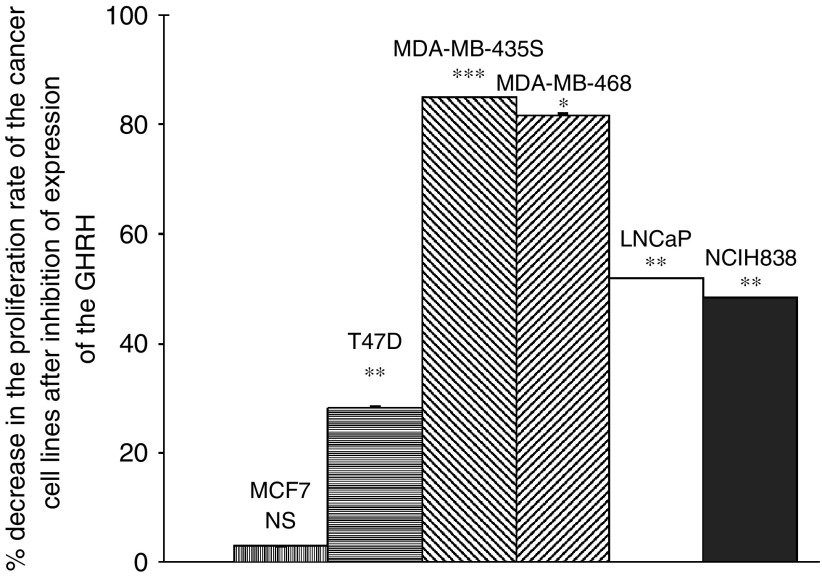
Graphic presentation of the decrease in the proliferation rate of the MBA-MB-468, MDA-MB-435s T47D, LNCaP and NCI H838 after the knocking down of the expression of the ectopic GHRH. MCF-7 breast cancer cell line was used as control. Vertical bars represent s.e.m. Data are representative of one experiment in triplicate in each case. Percentage decrease and signalling are expressed *vs* corresponding untreated cells. ^*^P<0.05, ^**^P<0.005, ^***^P<0.001.

The effective inhibition of the GHRH gene expression was confirmed by western blot. The results are seen in Figure 3. The GHRH expression in the transfected NCI H838, LNCaP, T47D cell lines was suppressed by 85, 85 and 92% respectively. The decreased proliferation rate of the MDA-MB-468 and MDA-MB-435s cell lines did not allow us to isolate protein from those cells during early cell passages. Since the silencing of the gene expression for GHRH lasts only for limited cell passages, the western blots of the proteins obtained in late cell passages show that the silencing of the GHRH gene expression was less effective for the two breast cancer cell lines in these passages (28 and 64% respectively). After the knocking down of GHRH expression, the proliferation rate of the T47D, MDA-MB-435s, MDA-MB-468, LNCaP, NCI H838 human cancer cell lines was decreased by 28.3, 85.9, 85.1, 51.8 and 48.4% respectively (Figure 4). The transfection of siRNA for GHRH did not influence the proliferation rate of MCF-7 cells. We also used control siRNA, which contains a scrambled sequence that does not lead to the specific degradation of any known cellular mRNA for transfection of all cell lines. No changes in the proliferation rate and no toxic effect were found in any cell lines.

### Effect of GHRH(1–29)NH_2_ on the proliferation of the knocked down cancer cell lines *in vitro*

**Figure 5:**
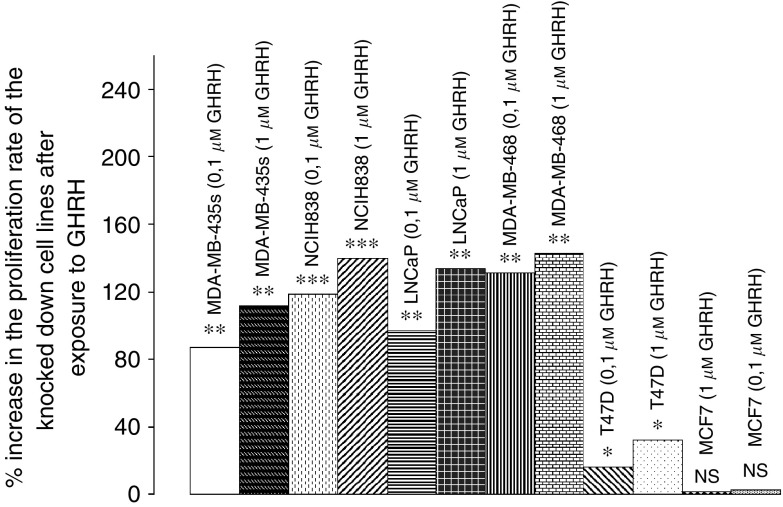
Effect of GHRH(1–29)NH_2_ at 0.1 and 1 *μ*M concentrations on the proliferation rate of the knocked down siMDA-MB-468, siMDA-MB-435s and siT47D breast, siNCI-H838 lung cancer and siLNCaP prostate cancer cell lines. MCF-7 breast cancer cell line was used as control. Data are representative of one experiment in triplicate. Percentage increase and significance is expressed *vs* knocked down cells cultured in the absence of GHRH. ^*^P<0.05, ^**^P<0.005, ^***^P<0.001.

When knocked down T47D, MDA-MB-486, MDA-MB-435s breast, LNCaP prostate and NCIH-838 lung cancer cell lines were exposed to 0.1 *μ*M. Growth hormone-releasing hormone (1–29)NH_2_ the proliferation rate of the cells was strongly stimulated by 16, 132, 87, 97, 119% respectively. The increase on the proliferation rate in response to 1 *μ*M GHRH was even greater, being 32, 143, 112, 134, 140% respectively. The GHRH did not affect the proliferation rate of MCF-7 breast cancer cell line, which was also transfected with siRNA for GHRH. The results are shown in Figure 5.

### Effect of GHRH(1–29)NH_2_ and GHRH antagonists on the proliferation of cancer cell lines *in vitro*

**Figure 6:**
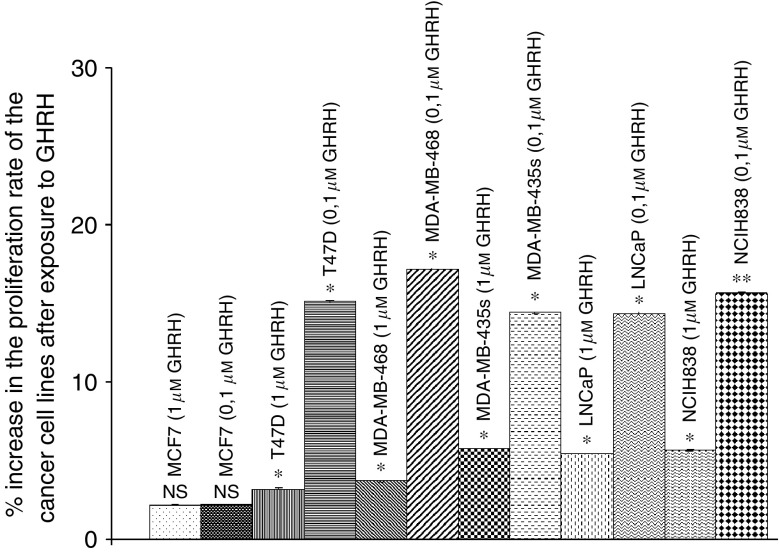
Effect of GHRH(1–29)NH_2_ at concentrations of 0.1 and 1 *μ*M on the proliferation rate of MCF-7, MDA-MB-468, MDA-MB-435s and T47D breast, lung (NCI-H838) and prostate (LNCaP) cancer cell lines. Vertical bars represent s.e.m. Data are representative of one experiment in triplicate. Percentage increase and significance are expressed *vs* cells cultured in the absence of GHRH. ^*^P<0.05, ^**^P<0.005.

T47D, MDA-MB-486, MDA-MB-435s breast, LNCaP prostate and NCIH-838 lung cancer cell lines cultured *in vitro* were exposed to two concentrations of GHRH(1–29)NH_2_ and GHRH antagonists MZ-5-156, JMR-132 and MZ-4-71. At the dose of 1 *μ*M GHRH (1–29)NH_2_ did not appreciably influence the proliferation rate of the cells, producing a change only of 1–5%. However GHRH (1–29)NH_2_ at 0.1 *μ*M concentration stimulated of the proliferation rate of the T47D, MDA-MB-468, MDA-MB-435s, LNCaP and NCI H838 cells by 15, 17, 14, 14, 16% respectively. The proliferation of MCF-7 cells (negative control) was not affected by GHRH at 0.1 and 1 *μ*M concentrations. The results are shown in Figure 6.

**Figure 7:**
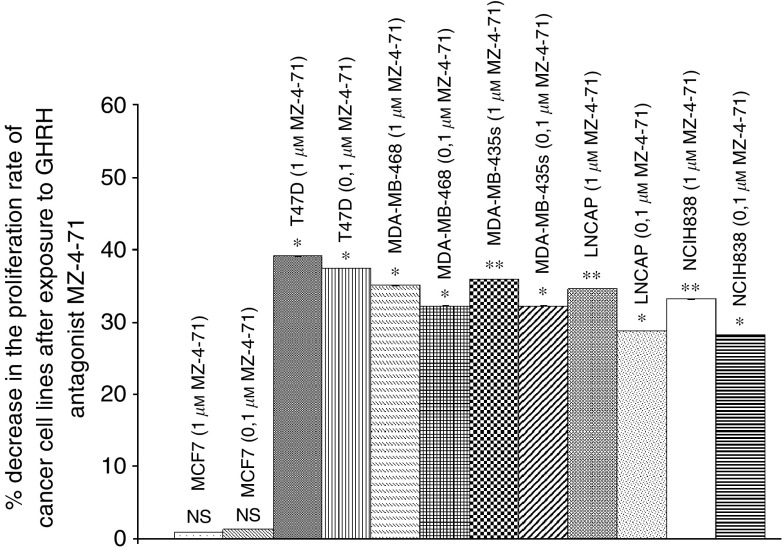
Effect of GHRH antagonist MZ-4-71 at two concentrations (0.1, 1 *μ*M) on the proliferation rate of MCF-7, MDA-MB-468, MDA-MB-435s and T47D breast, lung (NCI-H838) and prostate (LNCaP) cancer cell lines. Vertical bars represent s.e.m. ^*^P<0.001. Data are representative of one experiment in triplicate. Percentage increase and significance are expressed *vs* cells cultured in the absence of the antagonist. ^*^P<0.05, ^**^P<0.005.

Growth hormone-releasing hormone antagonist MZ-4-71 at the dose of 0.1 *μ*M decreased the proliferation of T47D, MDA-MB-468, MDA-MB-435s, LNCaP and NCI H838 cancer cell lines by 37, 29, 32, 29, 28% respectively. The results are presented in Figure 7. At the dose of 1 *μ*M MZ-4-71 produced a somewhat greater inhibition of proliferation of T47D, MDA-MB-468, MDA-MB-435s, LNCaP and NCI H838, the decreases being 39, 35, 36, 35 and 33% respectively. MZ-4-71 did not affect the proliferation of MCF-7 cells (negative control) (Figure 7).

**Figure 8:**
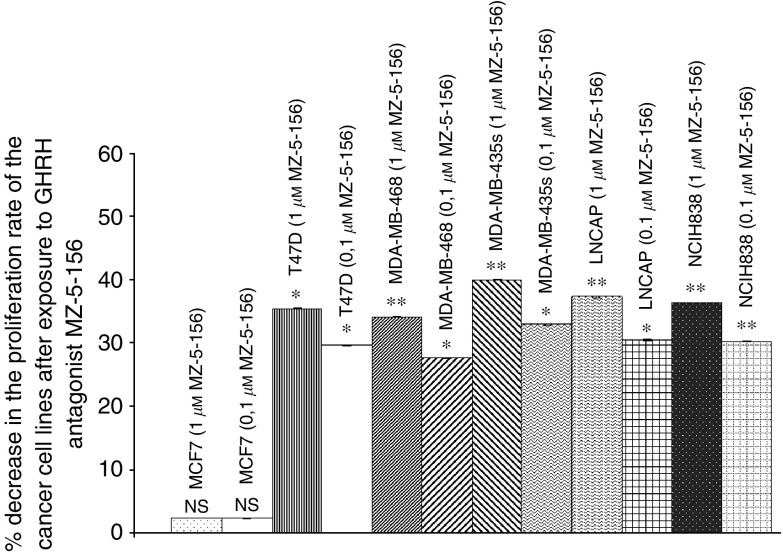
Effect of GHRH antagonist MZ-5-156 at two concentrations (0.1, 1 *μ*M) on the proliferation rate of MCF-7, MDA-MB-468, MDA-MB-435s and T47D breast, lung (NCI-H838) and prostate (LNCaP) cancer cell lines. Vertical bars represent s.e.m.^*^P<0.001. Data are representative of one experiment in triplicate % increase and significance are expressed *vs* cells cultured in the absence of the antagonist. ^*^P<0.05, ^**^P<0.005.

Similar inhibitory effects on proliferation were obtained with the other two antagonists. Thus GHRH antagonist MZ-5-156 at doses of 0.1 and 1 *μ*M reduced the proliferation of T47D, MDA-MB-468, MDA-MB-435s, LNCaP and NCI H838 cancer cell lines by 30–35, 28–34, 33–40, 30–37, and 30–36% respectively. Higher doses of the GHRH antagonist again caused a greater inhibition, indicated by the second set of numbers. The results are illustrated in Figure 8.

**Figure 9:**
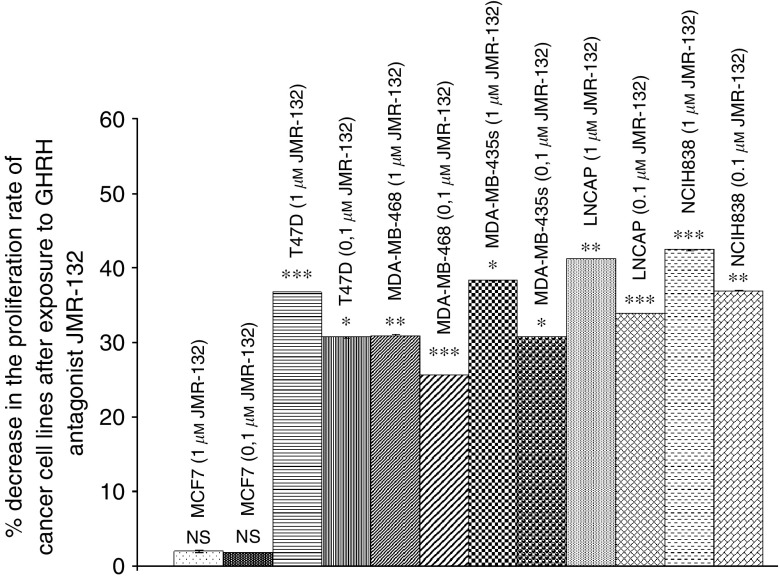
Effect of GHRH antagonist JMR-132 at two concentrations (0.1, 1 *μ*M) on the proliferation rate of MCF-7, MDA-MB-468, MDA-MB-435s and T47D breast, lung (NCI-H838) and prostate (LNCaP) cancer cell lines. Vertical bars represent s.e.m. ^*^P<0.001. Data are representative of one experiment in triplicate. Percentage increase and significance are expressed *vs* cells cultured in the absence of the antagonist. ^*^P<0.05, ^**^P<0.005, ^***^P<0.001.

The proliferation of T47Ds, MDA-MB-468, MDA-MB-435, LNCaP and NCI H838 cancer cell line was also inhibited by 31–37%, 26–31%, 31–38%, 34–41%, 37–42% respectively after exposure to GHRH antagonist JMR-132 at doses of 0.1 and 1 *μ*M. Higher doses of JMR-132 produced a greater inhibition, indicated by the second set of numbers. The results are shown in Figure 9.

## DISCUSSION

Growth hormone-releasing hormone and the major SV1 of the full length GHRH receptor are expressed in surgical specimens of diverse human cancers as well as in a various human cancer cell lines (reviewed in Schally and Varga, 2006). These findings led to the concept that GHRH may function as an autocrine growth factor in many human malignancies.

The precise role of the production of GHRH in the process of tumorigenesis and tumour progression has not been investigated previously. One way to elucidate the role of GHRH in the pathogenesis of cancer is to inhibit its gene expression. In the present study, we first examined the expression of pre-pro GHRH by western blot in human cancer cell lines. The band that reflects the production of the pre-pro GHRH appeared to have a molecular size of 45 kDa on the blots. The facts that this band disappeared in the knocked down cells and could not be detected in the negative control (MCF-7), together with the stimulation of the knocked down cells by exogenous GHRH, indicate that this band represents a precursor of GHRH (Othman *et al*, 2001). We also tried to identify bands corresponding to GHRH (1–44)NH_2_ by doing western blots for synthetic GHRH (1–44) NH_2_, but no corresponding signal was detected. Biologically active GHRH was detected by RIA in the conditioned medium of all the cancer cell lines, except for MCF-7.

We then evaluated the expression of the SV1 of GHRH receptor by western blot. The expression of the GHRH and its receptor SV1 in the breast, prostate and non-SCLC cancer cell lines examined suggested the presence of a stimulatory loop in those cells based on GHRH and its receptors. This raised the issue of establishing the role of GHRH in these cancer lines.

Small interfering siRNA for GHRH was used to elucidate the exact function of GHRH. The siRNA for GHRH is a pool of specially designed RNAs for knocking down the expression of human GHRH. After the transfection of the siRNA for GHRH, the proliferation rate of the MDA-MB-468 and MDA-MB-435s cancer lines was dramatically decreased by 85.1 and 85.9% respectively. The decreased proliferation, in combination with the fact that the silencing of the gene expression for GHRH lasts only for limited cell passages, did not allow us to isolate protein from these cells for western blot analysis during early cell passages. The isolation of the proteins obtained in late cell passages showed that the silencing of the GHRH gene expression was less effective in these passages.

In the case of the T47D cells the proliferation rate was decreased by 28.3%. The T47D cell line has the highest expression of SV-1 of the GHRH receptor with its ligand-dependent and independent activity. The inhibition of the gene expression for GHRH in T47D breast cancer cell line did not have the potent antiproliferative effect found in the other two breast cancer cell lines (MDA-MB-468 and MDA-MB-435S), possibly because the ligand independent activity of the SV1 continued to enhance the proliferation rate of the T47D cells (Kiaris *et al*, 2003; Barabutis *et al*, 2007).

Prostate cancer cell line LNCaP and non-SCLC cell line NCI H838 showed decreases in proliferation rates of 51.8 and 48.4% respectively after the inhibition of the GHRH gene expression. Both these cancer cell lines express SV-1 and because of its ligand-independent activity their proliferation rate continued to be stimulated in the absence of the intrinsic production of the GHRH.

The possible presence of GHRH and other related peptides such as VIP in the media of the cells that were tested cannot be excluded. Thus GHRH could keep acting as a growth factor and the proliferation rate of all the cancer cell lines that were assayed may not reflect the conditions of a total absence of GHRH.

MCF-7 breast cancer cells were also transfected with siRNA for knocking down the GHRH gene. This was done to test for any possible toxic effects, which could be related to the presence of the siRNA because MCF-7 line does not produce GHRH, its behaviour after the transfection had to remain the same. The transfection of the siRNA for GHRH did not influence the proliferation rate of the MCF-7 cells. Consequently the decreased proliferation rate of the breast, prostate and non-SCLC after the transfection was not due to toxic effects. The transfection of control siRNA did not lead to any changes in the proliferation of these cells.

In addition, we exposed the knocked down MDA-MB-468, MDA-MB-435s, T47D, LNCaP and NCI-H838 cancer cell lines to two different concentrations of GHRH (1–29)NH_2_. The proliferation rate of the cells was strongly stimulated by the addition of exogenous GHRH, not only at the concentration of 0.1 *μ*M but also at a concentration of 1 *μ*M. The silenced cancer cell lines, lacking intrinsic GHRH, were still able to respond to exogenous GHRH, confirming the absence of possible toxic effects related to the transfections. MCF7 cells transfected with siRNA for GHRH were also exposed to GHRH. The proliferation rate of the transfected MCF7 cells was not influenced by the addition of the exogenous GHRH.

We also investigated the effect of GHRH and GHRH antagonists at two concentrations in MCF-7, MDA-MB-468, T47D, MDA-MB-435s, LNCaP and NCI H838 cancer cell lines. The proliferation rate of the MCF-7 breast cancer cell line was not influenced by the presence of the GHRH or its antagonists, since MCF-7 does not express specific receptors for GHRH. At the dose of 0.1 *μ*M GHRH stimulated the proliferation rate of the cancer cell lines by 15–17%. However, at a dose of 1 *μ*M GHRH did not influence significantly the proliferation rate of cell lines. Thus, because of the possible presence of GHRH or other related peptides in the medium, as well as of GHRH secreted by the cells, it is likely that the corresponding signalling pathways were saturated after exposure to 1 *μ*M GHRH.

These breast, lung and prostate cancer cell lines were also exposed *in vitro* to two concentrations of GHRH antagonists MZ-4-71, MZ-5-156 and JMR-132. At concentrations of 0.1 and 1 *μ*M, the proliferation rate of these cells was decreased by 26–37% and 31–42% respectively.

The present study demonstrates the tumorigenic effect of GHRH in the human experimental tumour cell lines representative of leading cancers. Our work supports the concept that GHRH functions as growth factor in human cancers.

## Figures and Tables

**Table 1:** Production of GHRH in culture medium from human breast cancer (MCF-7, MDA-MB-468, MDA-MB-435S, T47D) prostate cancer (LNCaP) and non-small cell lung carcinoma (NCI H838) cell lines

	**GHRH (ng ml^−1^ medium) produced by CANCER CELL LINES**					
**Time (hour)**	**MCF-7**	**T47D**	**MDA-MB-468**	**MDA-MB-435s**	**LNCaP**	**NCI H838**
0	ND	ND	ND	ND	ND	ND
24	ND	1.518	0.637	0.675	0.202	0.303
48	ND	2.083	0.816	1.036	0.218	0.442
72	ND	0.266	0.649	1.442	0.208	0.689

ND=not detectable.

Aliquots of medium were subjected to RIA for the detection of GHRH at the indicated periods of time.
